# Matrix stiffness induces epithelial–mesenchymal transition and promotes chemoresistance in pancreatic cancer cells

**DOI:** 10.1038/oncsis.2017.54

**Published:** 2017-07-03

**Authors:** A J Rice, E Cortes, D Lachowski, B C H Cheung, S A Karim, J P Morton, A del Río Hernández

**Affiliations:** 1 Cellular and Molecular Biomechanics Laboratory, Department of Bioengineering, Imperial College London, London, UK; 2 Cancer Research UK Beatson Institute, Glasgow, UK

## Abstract

Increased matrix rigidity associated with the fibrotic reaction is documented to stimulate intracellular signalling pathways that promote cancer cell survival and tumour growth. Pancreatic cancer is one of the stiffest of all human solid carcinomas and is characterised by a remarkable desmoplastic reaction. Here we use mouse models, genetically engineered to recapitulate human pancreatic cancer, and several pancreatic cancer cell lines as a model to investigate the effect of matrix stiffness in epithelial–mesenchymal transition (EMT) and resistance to chemotherapeutics. We found that recapitulation of the fibrotic rigidities found in pancreatic cancer tissues promote elements of EMT, including increases in vimentin expression, decreases in E-cadherin expression, nuclear localisation of β-catenin, YAP and TAZ and changes in cell shape towards a mesenchymal phenotype. We also report that stiffness induces chemoresistance to paclitaxel, but not to gemcitabine, both commonly used therapeutics, suggesting that environmental rigidity underlies an aspect of chemoresistance.

## Introduction

Pancreatic ductal adenocarcinoma (PDAC) is the fourth most lethal cancer in the developed world, with >200 000 deaths per year across the world. Currently, the 5-year survival rate is <4%, and the cancer responds very poorly to chemotherapeutic agents.^1, 2^ PDAC tumours are usually detected at advanced stages due to rapid progression, with limited symptoms at early stages, meaning only 10% are operable.^2^ Tumours arise from ductal cells over an extended period, gradually accumulating mutations, from healthy pancreas to pancreatic intraepithelial neoplasia (PanIN), before full development into PDAC.^3^

The PDAC stroma is highly fibrotic due to desmoplasia, the deposition of a dense and crosslinked extracellular matrix (ECM), and is particularly pronounced in PDAC.^4, 5^ Fibrosis is an environmental property associated with risk of cancer development in liver cirrhosis and breast.^6^ The stiffness associated with desmoplasia can promote tumour malignancy across multiple organs, as the rigid stroma forces a tensional homeostasis with high levels of cell contractility to counteract the stiff environment, inducing intracellular signalling and malignant transformation.^7^ In breast cancer, tumorigenesis is associated with ECM stiffening and collagen crosslinking, which promotes the formation of integrin-containing focal adhesions at the cell membrane,^8^ leading to intracellular signalling involving extracellular signal–regulated kinase and ROCK-generated contractility, promoting a malignant phenotype.^7^ Additionally, matrix stiffness has been seen in hepatocellular carcinoma cells to regulate resistance to chemotherapeutics, including paclitaxel.^9^

The epithelial–mesenchymal transition (EMT) is a process in which cells become more motile through loss of cell–cell adhesion and their apical–basal polarity. This process is suggested to be vital for progression of PDAC,^10^ although controversial,^11^ as well as chemoresistance.^12^ EMT is a multifaceted transition and is characterised through changes in cell morphology and the behaviour of many proteins, including vimentin, E-cadherin, β-catenin,^13^ YAP and TAZ.^14^ Many elements of EMT have been observed in pancreatic cancer, including elevated expression of YAP and TAZ^15^ and increased nuclear YAP localisation and activity.^16^ Stiffness is associated with EMT in other cancers, such as in breast cancer, where a stiff ECM induces elements of EMT through mechanotransduction.^17^ Mechanical activation of β-catenin has been observed for *in vivo* mouse colon,^18^ and vimentin organisation has been seen to be altered by the *in vivo* mechanical environment.^19^ YAP and TAZ, transcription factors known to be associated with EMT, have also emerged recently as key players that control induction of fundamental cell processes in response to ECM stiffness.^20^

Resistance to chemotherapeutic drugs has many suggested causes, including cancer cells with intrinsic resistance mechanisms, as well as stromal cells maintaining a desmoplastic microenvironment that promotes cancer cell resistance by providing an environment that hampers drug delivery.^21^ EMT in PDAC, as induced through Snail or Twist pathways, has been suggested to be unnecessary for invasion and metastasis, with knockout of either the Snail or Twist effectors not affecting tumour dissemination but instead playing an important role in chemoresistance to the antiproliferative agent gemcitabine.^11^ Most chemotherapies for pancreatic cancer are based on gemcitabine.^21^ Loss of the EMT marker TAZ in breast cancer cells impairs their chemoresistance,^22, 23^ and TAZ is known to be upregulated in pancreatic cancer cells.^15^

Owing to the highly fibrotic nature of PDAC and its association with poor survival, we investigate this form of cancer for mechanical induction of a malignant phenotype. First, we characterise the mechanics of healthy pancreas, PanIN and PDAC tissues. We then show that recapitulation of the fibrotic rigidities can promote elements of EMT in pancreatic cancer cell lines, including increases in vimentin expression, decreases in E-cadherin expression, nuclear localisation of β-catenin, YAP and TAZ and changes in cell shape towards a mesenchymal phenotype. This indicates not only the role of stiffness in induction of the mesenchymal phenotype but also the plasticity and non-discrete nature of the transition. We also report that stiffness induces chemoresistance to paclitaxel, but not to gemcitabine, suggesting that environmental rigidity underlies an aspect of chemoresistance.

## Results

### PDAC progression shows increased tissue tension and alignment and thickening of collagen fibres

To characterise in detail the changes that occur in the collagen architecture and tissue mechanics in the pancreas during the transition from healthy pancreas to premalignant lesions to PDAC, we used pancreas from (i) C (Pdx1-Cre) mice for normal pancreas,^24^ (ii) KC PanIN mice (Pdx1-Cre; LSL-Kras^G12D/+^) for premalignant lesions and (iii) KPC mice (Pdx-1 Cre, LSL-Kras^G12D/+^, LSL-Trp53^R172H/+^) for PDAC pancreas.^25^ PDX-Cre driver lines are used in these mouse models to conditionally activate the target genes, with the transcription factor PDX present in all pancreatic cells.^26^ Mutations in the tumour-suppressor gene p53 and activating mutations in the Kras oncogene are commonly used to recapitulate pancreatic cancer phenotypes in mouse models^27^ and are seen frequently in human pancreatic cancers, with p53 mutations in 75% and Kras mutations in 95% of cancer samples.^28^

**Figure 1:**
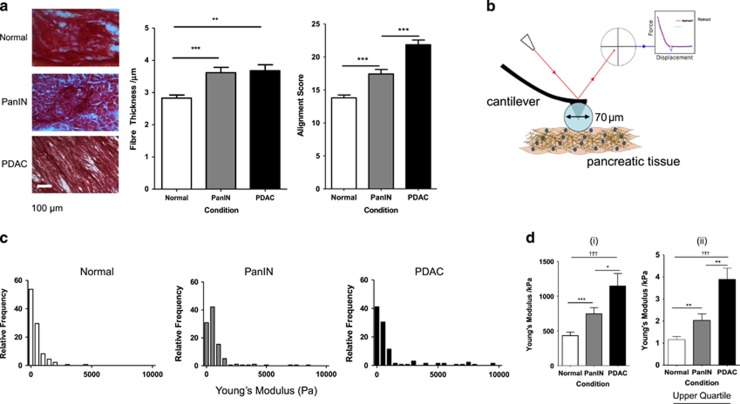
*In vivo* characterisation of extracellular changes during PDAC progression. (**a**) Sirius Red staining of 10 μm-thick sections of mouse pancreatic tissue for normal pancreas, PanIN and PDAC. Fibre thickness is quantified through BoneJ analysis (normal, *n*=41; PanIN, *n*=48; PDAC, *n*=71) and alignment score through Radial Summation Alignment Analysis of 75 × 75 μm^2^ regions of interest (normal, *n*=65; PanIN, *n*=45; PDAC, *n*=52). Values represent mean±s.e.m. **P<0.01, ***P<0.0001. (**b**) Schematic diagram of AFM determination of Young’s modulus of pancreatic tissue. A laser reflected off a cantilever is deflected by deformation of a cantilever with a 70 μm glass bead attached. This produces a force curve that is fitted to the Hertz model for a Young’s modulus value. (**c**) Histograms showing the range of local Young’s modulus measurements across pancreatic tissue conditions: normal (*n*=132), PanIN (*n*=131), and PDAC (*n*=175). (**d**) Bar graph from histograms in panel (**c**), representing the whole data set (i) and the upper quartile (ii) data points from histograms in panel (**c**), representing the maximum rigidities present in the tissue. Normal, *n*=31; PanIN, *n*=33; PDAC, *n*=41. Values represent mean±s.e.m. *P<0.05, **P<0.001, ***P<0.0001 for unpaired Mann–Whitney test. ^†††^*P*<0.0001 for Kruskal–Wallis test.

The amount of collagen-I is known to be increased in the fibrotic stroma of PDAC compared with normal pancreas, and this promotes cell survival and proliferation.^16, 29^ Thick collagen fibres are indicative of poorly differentiated PDAC tumours and short survival of patients.^16^ Using Sirius Red staining of 10 μm-thick sections of tissue, we observe that fibre thickness differs significantly from normal pancreas (2.8 μm) to the intermediate PanIN stage (3.6 μm) and PDAC (3.7 μm) (Figure 1a). Fibre thickness was calculated with the ImageJ plugin BoneJ.^30^ The thickening of fibres therefore occurs at an early stage of cancer development before full progression to PDAC.

Alignment of collagen-I fibres has been observed in PDAC samples^31^ and is known to promote metastasis in breast cancer, possibly by changing matrix stiffness and/or reducing spatial impedance to enhance migration.^32^ The analysis technique used here produces a two-dimensional Fourier transform of the original image and performs radial summation (summing pixel intensities along each radial angle) to assess the anisotropy of the Fourier transform, which is related to the alignment of pixels in the original image.^33^ Taking images across the entire section, collagen fibres appear to be loosely organised in the normal pancreas, showing limited order and alignment, whereas fibres in PDAC appear to show high levels of organisation and alignment, in agreement with previous studies.^31^ PanIN shows small regions of alignment but is overall disorganised. Radial summation alignment analysis^33^ has been used to quantify these changes for 75 × 75 μm^2^ regions of interest, indicating significant increases in fibre alignment during PDAC progression (Figure 1a). Estimation of collagen organisation by Sirius Red alone does not comprehensively detail fibre organisation, as other weakly birefringent materials such as keratin and fibrin may also be stained and fibres parallel to the light transmission axis are not visualised when linearly polarised light is used.^34^

Tissue stiffness is associated with tumour malignancy, and the Young’s moduli of pancreatic tissue samples were assessed through atomic force microscopy (AFM) (Figure 1b), indicating an increase in stiffness between a PanIN and a PDAC mouse model, at a micron scale.^16^ AFM here is used at a larger scale with a bead of 70 μm diameter for direct comparison with the regions of collagen fibre analysis. This evaluation shows a heterogeneity in tumour stiffness (Figure 1c), an observation previously seen in breast cancer.^35^ Histograms showing the range of stiffnesses show a skew to softer regions (<1 kPa) in all conditions, but with progression from healthy pancreas to PanIN to PDAC, this skew is less prevalent, demonstrating that stiffness increases with fibrosis and suggesting altered mechanotransduction. Low stiffness regions are present in all samples, suggesting that ECM remodelling during fibrosis occurs at a local scale and to specific elements of the tissue.

The mean values for Young’s modulus (Figure 1d(i)) differ significantly between normal and PanIN (*P*<0.0001) and between normal and PDAC (*P*=0.0423), though no significant difference is observed between PanIN and PDAC (*P*=0.0751). A one-way non-parametric analysis of variance determines that the medians are significantly different between conditions (*P*<0.0001). Mechanosensitivity is likely dominated by the stiffer regions of the tissue, and therefore the upper quartile measurements of the data set are also compared (Figure 1d(ii)), showing a significant increase in Young’s modulus from healthy pancreas (1 kPa) to PanIN (2 kPa) to PDAC (4 kPa). These upper quartile mean stiffness values were taken forward into *in vitro* experiments.

### Elements of EMT are induced *in vitro* in pancreatic cancer cells by PDAC-relevant rigidities

Based on the *in vivo* stiffness variations observed with AFM, pancreatic cancer cell lines were cultured on polyacrylamide gels of tunable rigidity to observe molecular changes associated with EMT. The stiffness values of 1, 4 and 25 kPa were created using differing concentrations of acrylamide/bisacrylamide to represent healthy pancreas, PDAC and an extreme stiffness, respectively. Acrylamide/bisacrylamide ratios were used to produce gels of a specific Young’s modulus and verified by AFM (Supplementary Figure S1), before being coated with fibronectin to produce the same surface ligand profile.

Three pancreatic cancer cell lines were used, originating from different stages of cancer development. BxPC-3 cells represent a highly epithelial phenotype, derived from a patient with no observed metastases and show mutations in the gene for p53,^36^ and high expression of the marker E-cadherin, a protein involved in cell–cell adhesion^37^ and associated with the epithelial phenotype.^38^ AsPC-1 cells represent an intermediate phenotype from a patient with some metastasis^36^, show faster proliferation than BxPC-3 cells and have mutations in the genes for Kras and p53.^36^ Suit2-007 cells represent a highly mesenchymal phenotype, causing spontaneous metastasis in nude mice xenografts.^39^ Characteristic mesenchymal phenotypic markers are assessed to demonstrate EMT across the cell population: increases in vimentin expression, decreases in E-cadherin expression, increases in nuclear localisation of β-catenin, and a more elongated cell shape.^40^

**Figure 2:**
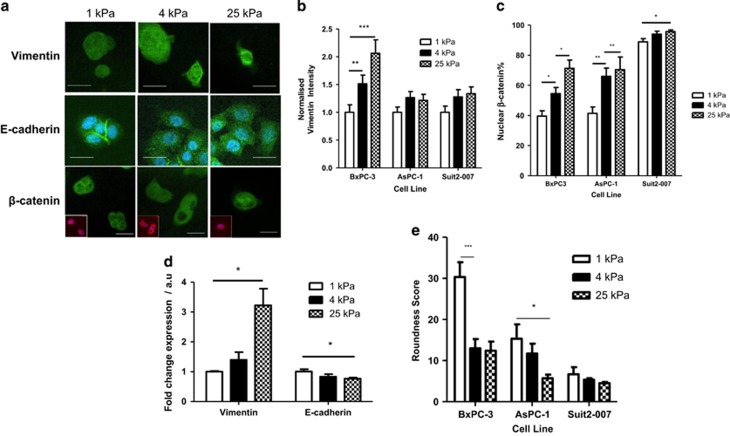
Role of matrix stiffness in EMT induction. (**a**) Vimentin, E-cadherin and β-catenin immunofluorescence images of BxPC-3 cells on matrices of varying stiffness. For β-catenin, main image shows merge of marker staining (green), top right inset is marker staining and bottom left inset is phalloidin staining (red) of actin cytoskeleton to show cell shape and DAPI (blue). Scale bar=25 μm. (**b**) Normalised vimentin intensity (corrected cell total fluorescence). For 1, 4 and 25 kPa, respectively, BxPC-3, *n*=33, 39, 31; AsPC-1, *n*=31, 35, 29; Suit2-007, *n*=41, 33, 45. Values represent mean±s.e.m. **P<0.01, ***P<0.0001. (**c**) Nuclear localisation percentage for β-catenin and YAP. For 1, 4 and 25 kPa, respectively, BxPC-3, *n*=13, 9, 13; AsPC-1, n 10, 11, 7; Suit2-007, *n*=11, 11, 12. Values represent mean±s.e.m. *P<0.05, **P<0.01. (**d**) Expression of EMT markers vimentin and E-cadherin at the mRNA level. Values represent mean±s.e.m. For an unpaired *t*-test, *P<0.05. *N* values for each protein at 1, 4 and 25 kPa, respectively, vimentin: 4, 6, 5; E-cadherin: 3, 6, 4. (**e**) Quantification of roundness for cells grown on matrices of varying rigidities. For 1, 4 and 25 kPa, respectively, BxPC-3, *n*=13, 15, 15; AsPC-1, *n*=19, 19, 15; Suit2-007, *n*=17, 13, 13. Values represent mean±s.e.m. *P<0.05, ***P<0.001.

All cell lines, when grown on gels with a stiffness of 1, 4 and 25 kPa, showed increases in vimentin expression with gel rigidity, as observed by immunofluorescence (Figure 2a, Supplementary Figure S2A), PCR (Figure 2d) and western blotting (Supplementary Figure S3) were also used for BxPC-3 cells. With vimentin immunofluorescence intensity normalised to 1.0 for the 1 kPa data set for each cell line, the highly epithelial cell line BxPC-3 showed high sensitivity to matrix rigidity with vimentin expression increasing by a factor greater than that observed in the more mesenchymal cell lines, AsPC-1 and Suit2-007 (Figures 2a and b). Localisation of the cell adhesion-associated molecule β-catenin to the nucleus is indicative of EMT and is here observed by immunofluorescence (Figures 2a and c) to become localised to the nucleus with increasing stiffness in all cell lines, with the more epithelial line BxPC-3 once again showing higher sensitivity to stiffness-induced EMT than the mesenchymal Suit2-007 line. Cell shape is observed to change with stiffness also, with the parameter of cell roundness (calculated from the variance of the radii of the cell) decreasing with stiffness, with BxPC-3 showing the highest sensitivity (Figure 2e).

The highly sensitive cell line BxPC-3 was also characterised for E-cadherin expression, as well as the additional markers of (i) loss of intracellular junctions and (ii) abundance of cytoskeletal filaments, both of which have been previously classified as minor criteria for EMT.^13^ E-cadherin, a marker for the epithelial phenotype, is seen through immunofluorescence and PCR in BxPC-3 cells to decrease with increasing stiffness (Figures 2a and d), showing loss of cell–cell adhesion. The percentage of BxPC-3 cells that do not show clustering behaviour increases with stiffness, denoting loss of cell–cell junctions and subsequent individualisation (Supplementary Figure S2B), though this measure is only a surrogate marker for changes in cell adhesion.

Changes in extracellular stiffness are expected to induce a tensional homeostasis in which the cytoskeleton is remodelled and reinforced to match the force provided by the environment. This is observed through increases in F-actin levels (Supplementary Figure S2C). Phalloidin staining was used for F-actin quantification as this bicyclic heptapeptide binds specifically to F-actin filaments without binding to the monomeric G-actin.^41^ F-actin intensity is used here as a surrogate marker for cell contractility, and further experiments such as traction force microscopy would be needed to document changes in cell force.

The plasticity of EMT is therefore revealed, as gradual increases in stiffness can progressively promote mesenchymal behaviour rather than just a binary switch between the epithelial and mesenchymal phenotypes.

### Matrix rigidity promotes YAP/TAZ nuclear localisation

YAP and TAZ are functionally redundant homologous transcriptional activators that can promote oncogenic transformations, such as EMT.^42^ YAP/TAZ is activated by dephosphorylation and in this state can shuttle from the cytoplasm into the nucleus to bind to transcription factors such as TEAD and promote gene expression. Phosphorylation of YAP by LATS1/2 on Ser127 is inhibitory and prevents nuclear localisation through cytoplasmic sequestration by proteins, such as 14-3-3 and DVL.^43^ YAP and TAZ nuclear localisation has been shown to be influenced by mechanical forces,^20^ and their expression is increased in patients with either PDAC or chronic pancreatitis.^15, 44^

**Figure 3:**
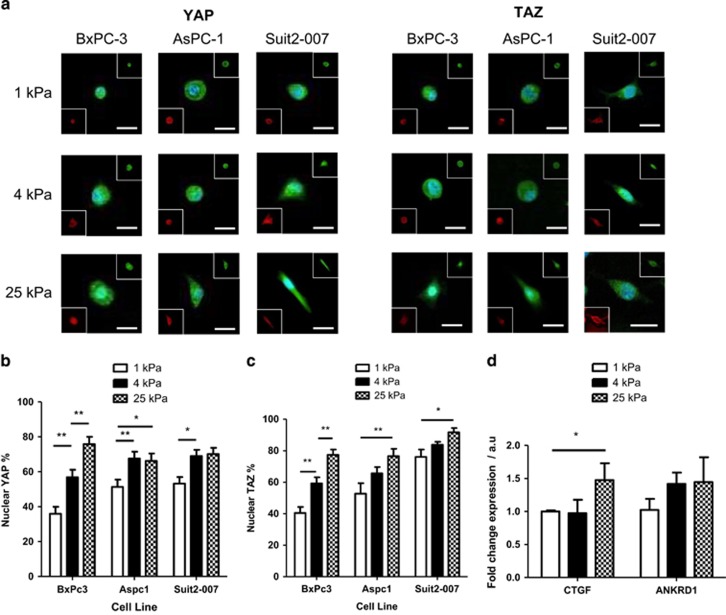
Localisation of YAP/TAZ in response to matrix stiffness. (**a**) Changes in YAP and TAZ localisation to the nucleus in BxPC-3, AsPC-1 and Suit2-007 cell lines, as shown by immunofluorescence, with increasing matrix stiffness. For β-catenin, main image shows merge of marker staining (green) and DAPI (blue) and bottom left inset is a merge of phalloidin staining (red) of actin cytoskeleton to show cell shape and DAPI. Scale bar=25 μm. (**b**) Nuclear localisation percentage of YAP. For 1, 4 and 25 kPa, respectively, BxPC-3, *n*=22, 22, 16; AsPC-1, *n*=14, 14, 13; Suit2-007, *n*=12, 15, 18. (**c**) Nuclear localisation percentage of TAZ. For 1, 4 and 25 kPa, respectively, BxPC-3, *n*=19, 24, 20; AsPC-1, *n*=20, 19, 21; Suit2-007, *n*=13, 11, 10. Values represent mean±s.e.m. *P<0.05, **P<0.01. (**d**) Expression of YAP target genes CTGF and ANKRD1 at the mRNA level. Values represent mean±s.e.m. For an unpaired *t*-test, *P<0.05. *N* values for each protein at 1, 4 and 25 kPa, respectively; CTGF: 6, 6, 4; ANKRD1: 3, 5, 3.

For the three different pancreatic cancer cell lines, both YAP and TAZ localisation significantly increase with gel rigidity, with the highest mechanosensitivity in BxPC-3 cells, the most epithelial of the cell lines (Figures 3a–c), showing the same trend as other mesenchymal markers. YAP localisation reaches a maximum of 70–75%, whereas TAZ localisation is slightly higher at around 80% for BxPC-3 and AsPC-1 cells, reaching an increased value of 90% in the highly mesenchymal Suit2-007 cells. YAP and TAZ vary in structure^45^ and these slight differences may underlie the minor difference in localisation percentage as part of the stiffness response. The expression of the YAP target genes CTGF and ANKRD1^20^ are seen to increase with stiffness for BxPC-3 cells through PCR (Figure 3d), confirming that YAP/TAZ activity is affected by stiffness.

Though matrix stiffness can promote localisation of YAP and TAZ, this phenotype is not dominant over known inhibitors of YAP/TAZ localisation (Supplementary Figure S4). Adrenaline stimulates G-protein-coupled receptor signalling, leading to phosphorylation and deactivation of YAP/TAZ,^46^ and 10 μM adrenaline is observed here to inhibit YAP localisation even under high stiffness conditions. Additionally, blebbistatin, which inhibits actomyosin contraction and therefore prevents YAP/TAZ nuclear localisation,^47^ is observed to inhibit YAP/TAZ localisation at a concentration of 50 μM across the range of rigidities tested.

### Matrix stiffness promotes chemoresistance to paclitaxel but not gemcitabine

BxPC-3 cells show the highest sensitivity to matrix stiffness in induction of EMT, and we used these cells to assess the role of stiffness in inducing chemoresistance. Resistance to chemotherapeutic drugs underlies the malignancy of PDAC, with EMT known to induce a resistant phenotype.^21^ Multiple cytotoxic drugs are used in PDAC chemotherapies, including gemcitabine^21^ and paclitaxel.^48^ Gemcitabine is a nucleoside analogue used in treatment of metastatic pancreatic cancer^49^ where it competes against deoxycytidine triphosphate and cytidine triphosphate for incorporation into DNA and RNA, respectively.^50^ *In vivo* EMT has been observed to protect pancreatic cancer cells from the antiproliferative effects of gemcitabine.^11^ Paclitaxel is a taxane, which binds to, and stabilises, microtubules to suppress their dynamics and subsequently inhibit mitosis^51^ and is used as a treatment for metastatic pancreatic cancer in an albumin-bound form.^52^ Treatment of patients with paclitaxel has been shown to decrease tumour stiffness *in vivo*.^53^ Resistance to paclitaxel and mesenchymal phenotype are uncorrelated among pancreatic cell lines,^54^ though EMT is observed to promote paclitaxel resistance for *in vitro* breast cancer cell lines.^55^

**Figure 4:**
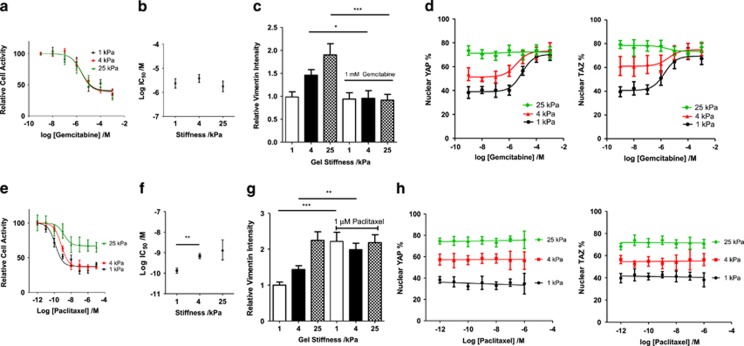
The role of matrix stiffness in gemcitabine and paclitaxel chemoresistance. (**a**) Dose–response curve for BxPC-3 cell viability with increasing concentrations of gemcitabine. For 1, 4 and 25 kPa, respectively, total *n*=81, 74, 65. Values represent mean±s.e.m. (**b**) IC_50_ values from dose–response curves in panel (**a**). Values represent mean±s.e.m. (**c**) Normalised vimentin intensity (corrected cell total fluorescence). For 1, 4 and 25 kPa, respectively, for control conditions, *n*=23,20,20. For 1, 4 and 25 kPa, respectively, for 1 mM gemcitabine conditions, *n*=18, 13, 15. Values represent mean±s.e.m. *P<0.05, ***P<0.001. (**d**) Changes in population nuclear YAP percentage. For 1, 4 and 25 kPa, respectively, total *n*=76, 74, 78. Changes in population nuclear TAZ percentage. For 1, 4 and 25 kPa, respectively, total *n*=72, 76, 68. Values represent mean±s.e.m. (**e**) Dose–response curve for BxPC-3 cell viability with increasing concentrations of paclitaxel. For 1, 4 and 25 kPa, respectively, total *n*=58, 67, 54. Values represent mean±s.e.m. (**f**) IC_50_ values from dose–response curves in panel (**e**). Values represent mean±s.e.m. (**g**) Normalised vimentin intensity (corrected cell total fluorescence). For 1, 4 and 25 kPa, respectively, for control conditions, *n*=42, 39, 23. For 1, 4 and 25 kPa, respectively, for 1 mM gemcitabine conditions, *n*=32, 20, 30. Values represent mean±s.e.m. **P<0.01, ***P<0.001. (**h**) Changes in population nuclear YAP percentage. For 1, 4 and 25 kPa, respectively, total *n*=58, 56, 66. Changes in population nuclear TAZ percentage. For 1, 4 and 25 kPa, respectively, total *n*=77, 59, 65. Values represent mean±s.e.m.

For rigidities of 1, 4 and 25 kPa, the response of BxPC-3 cells to gemcitabine was unaffected by matrix rigidity (Figures 4a and b). The IC_50_ values for 1, 4, and 25 kPa were 2, 4 and 2 μM, respectively, and not significantly different from each other (*P*=0.6, extra sum of squares F-test). Vimentin intensity under control and 1 mM gemcitabine conditions was assessed (Figure 4c), showing that the drug prevented the mesenchymal phenotype from persisting which may underlie the similarity in response of these cells under increasing rigidities. Moreover, both YAP and TAZ localisation were observed to increase with an increasing concentration of gemcitabine, showing 70–80% localisation in all rigidities at a gemcitabine concentration of 1 mM (Figure 4d). This apparent selection for mesenchymal cells is inconsistent with the observed alterations in vimentin, tentatively suggesting an alternative process in which gemcitabine, by preventing transcription through its nucleoside analogue activity, deregulates the LATS1/2 feedback loop^56^ in which transcriptional activation of LATS1/2 by YAP/TAZ is necessary. Further experiments involving assessment of LATS1/2 activation are required to evaluate this hypothesis.

In contrast, 4 and 25 kPa matrices are observed to promote increased levels of chemoresistance of BxPC-3 cells to paclitaxel when compared with the 1 kPa culture condition (Figures 4e and f). IC_50_ values were calculated as the paclitaxel concentration that produced 50% of the maximum inhibition response, which was not 100% inhibition for any of the cell conditions. Cells on a 1 kPa gel show an IC_50_ value of 0.1 nM (log IC_50_=−9.9), significantly different (*P*=0.0004) than the 0.7 nM value (log IC_50_=−9.2) for the 4 kPa condition. Furthermore, the 25 kPa condition has a higher IC_50_ value of 1.4 nM (log IC_50_=−8.9). The minimum level of cell viability achieved by paclitaxel exposure is around 37% for 1 and 4 kPa conditions and significantly higher (*P*<0.0001) at 67% for the 25 kPa condition. The partial mesenchymal phenotype therefore achieved at 4 kPa (Figure 2) appears to be correlated with paclitaxel sensitivity, and this chemoresistance is further potentiated at the 25 kPa condition. Paclitaxel treatment of 1 μM is also observed to promote an increase in the intensity of the mesenchymal marker vimentin (Figure 4g) across all three rigidities, suggesting that the cells that survive the inhibitory concentration of paclitaxel are cells with a mesenchymal phenotype. It can therefore be speculated that the mesenchymal phenotype, present in an increasing number of cells with increasing stiffness, is conferring resistance to paclitaxel. In a further difference to gemcitabine, YAP/TAZ levels do not change with inhibitory concentrations of paclitaxel (Figure 4h), suggesting that, for 1 and 4 kPa at least, drug-induced cell death does not discriminate based on YAP/TAZ localisation.

## Discussion

In this study, we characterise the mechanical and structural properties of PDAC tissues following development from a healthy state and assess the role of the matrix rigidity component in promoting the malignant phenotype of EMT and chemoresistance in pancreatic cancer cells *in vitro*. Matrix rigidity is observed to be able to promote progression from the epithelial towards the mesenchymal phenotype in pancreatic cancer cell lines and allows insight into the role of EMT in resistance to the chemotherapeutic agents, gemcitabine and paclitaxel. These findings begin to specifically examine the role of matrix stiffness in the PDAC microenvironment in tumour malignancy and allude to an *in vivo* environment in which multiple external components act synergistically to induce malignant properties.

Development of PDAC from healthy pancreas involves many changes in the ECM and its properties. These include increases in tissue stiffness, as consistent with previous studies,^16^ as well as alterations in ECM organisation comprising increases in alignment and thickness of collagen-I fibres. The region of interest sizes were chosen to be large enough to capture the heterogeneous nature of the tissue, yet localised enough to detect specific changes to the ECM. The use of non-subjective quantitative analysis tools for specific quantification of aspects of collagen organisation using recently developed algorithms^30, 33^ adds further characterisation of the desmoplasia involved and removes the possibility of bias and subjectivity in conclusions. This is particularly relevant considering that recent studies have demonstrated that bulk collagen is a poor predictor of disease progression in PDAC.^16^

Following this *in vivo* characterisation, polyacrylamide gels were used to recapitulate the values of tissue rigidities observed in healthy and diseased pancreas to determine the specific role of matrix rigidity in promoting the malignant cell processes of EMT and chemoresistance in pancreatic cancer cell lines. EMT is characterised by particular changes of marker proteins and cell morphology, which were observed to show gradual increases in mesenchymal behaviour following culture on gels of increasing rigidity, for multiple pancreatic cancer cell lines. The sensitivity of EMT induction to matrix stiffness correlates to the original phenotype of the cells. The highly epithelial cell line BxPC-3 shows the highest sensitivity, the highly mesenchymal cell line Suit2-007 shows a lower responsiveness and the intermediate cell line AsPC-1 shows an intermediate receptivity. The gradual nature of the transition observed here confirms its plasticity under these *in vitro* conditions. In contrast, YAP, TAZ and β-catenin localisation events occur within individual cells in a binary manner, and this suggests that other mechanotransduction pathways, such as the AKT or mitogen-activated protein kinase pathways that are known to promote EMT in response to stiffness in other cancers,^40^ should be investigated for further understanding of mechanosignalling and EMT.

The ability of cancer cells to escape the cytotoxic effects of therapeutics drugs is fundamental for cancer progression. Multiple drugs are used in PDAC treatment with differing mechanisms of action, and here we observe a marked dependence of the drug mechanism of action in determining whether the stiffness component of PDAC is responsible for inducing chemoresistive properties. *In vivo* EMT is known to promote resistance to gemcitabine, a nucleoside analogue that inhibits DNA synthesis and transcription.^54^ Here we report that *in vitro* gemcitabine resistance is unchanged with increased rigidity, despite also observing that gel rigidity can promote elements of EMT. Gemcitabine treatment is also seen to cause YAP/TAZ nuclear localisation to a percentage seen for 25 kPa gels, likely due to deregulation of the LATS1/2-mediated negative feedback loop^56^ by preventing transcription of LATS1/2. This would lead to YAP/TAZ dephosphorylation and nuclear localisation but without further EMT induction. This is corroborated by the observation that vimentin expression on high stiffness gels reverts to a level as seen on 1 kPa gels with 1 mM concentrations of the drug. As gemcitabine is a nucleoside analogue that inhibits DNA replication, it would also likely inhibit transcription and therefore inhibit expression of mesenchymal markers and maintenance of the phenotype.

Paclitaxel, a taxane that stabilises microtubules and therefore prevents mitosis, has shown potential for PDAC treatment, as it is one of the few drugs that has significantly improved the survival rates in patients when combined with nucleoside analogues, such as gemcitabine or platinum derivatives.^52^ In contrast to gemcitabine, cells grown on 4 and 25 kPa gels show increases in resistance to paclitaxel compared with the 1 kPa conditions. The 4 kPa condition therefore, representing PDAC stiffness, is an intermediate stage in the transition that provides some drug-resistive properties and in which cells with or without YAP/TAZ nuclear localisation respond in a similar manner to the presence of paclitaxel. Gemcitabine and an albumin-bound form of paclitaxel have been shown in combination to promote overall survival for patients with metastatic PDAC in a randomised phase III trial,^57^ and their effectiveness may be due to the potent combination of abrogation of the mesenchymal phenotype and toxicity to epithelial cells. This is speculated to act in addition to the known ability of paclitaxel to potentiate gemcitabine toxicity *in vivo* by decreasing levels of cytidine deaminase,^58^ which dephosphorylates and thus inactivates gemcitabine.^59^ Though stiffness is seen here to induce paclitaxel resistance *in vitro*, paclitaxel has been observed to decrease tumour stiffness *in vivo*,^53^ and it is yet to be determined whether the paclitaxel-resistant phenotype induced by stiffness can persist in a lower stiffness environment.

**Figure 5:**
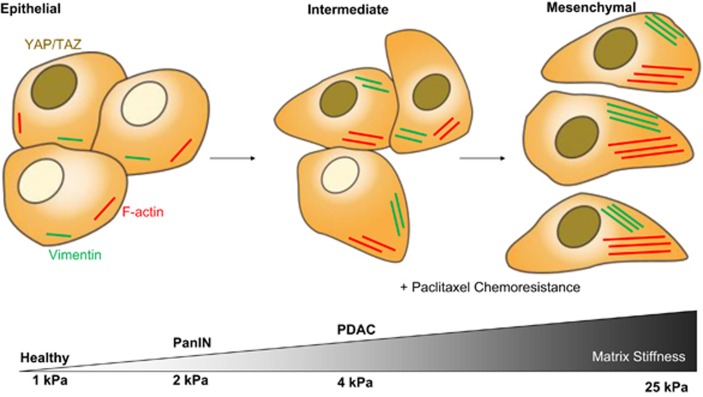
Diagram of stiffness-induced progression of the EMT. The proposed role of stiffness in promoting progression through EMT in pancreatic cancer cells and its relevance to *in vivo* measurements of tissue stiffness with PDAC progression.

The characterisation here of how stiffness promotes different elements of EMT to different degrees, as shown in Figure 5, has great relevance for clinical understanding of PDAC development. Therapeutics that seek to the disrupt the mechanosensing of cells within the tumour may benefit from an understanding of the specific role of stiffness in EMT induction and the possible role of stiffness in combination with other variables associated with the PDAC microenvironment. It is therefore vital for further studies to be conducted that assess whether matrix stiffness can act synergistically with other microenvironmental cues. For example, the *in vitro* mesenchymal phenotype promoted by matrix rigidity is seen here to be ineffective in gemcitabine resistance, whereas the *in vivo* mesenchymal phenotype promotes resistance.^11^ This is likely due to other *in vivo* factors inducing further elements of EMT, perhaps a further induced transition to a more stable mesenchymal state in which cells can prevent the action of gemcitabine to a greater degree.

## Materials and methods

### Mouse tissues

Mouse tissues for healthy pancreas (Pdx-1-Cre), PanIN (Pdx1-Cre; LSL-Kras^G12D/+^) and PDAC (Pdx1-Cre; LSL-Kras^G12D/+^ LSL-Trp53^R127H/+^) were obtained from Dr Jennifer Morton at the Beatson Institute in Glasgow, UK in the form of Optimal Cutting Temperature (OCT)-embedded tissues and frozen tissue samples. All studies were conducted in compliance with the UK Home Office guidelines under license and approved by the local ethical review committee.

### Histology

OCT-embedded samples were sectioned with a cryostat. These formalin-fixed OCT-embedded 10 μm sections were stained with Sirius Red in picric acid and viewed by brightfield microscopy. Alignment analysis was carried out on non-overlapping regions of 75 × 75 μm^2^ size as in Robinson *et al.*^33^ BoneJ analysis^30^ was used for fibre thickness quantification.

### Atomic force microscopy

Stiffness was measured by AFM with a JPK Nanowizard-1 (JPK Instruments, Berlin, Germany) in force spectroscopy mode, mounted on an inverted optical microscope (IX-81; Olympus, Tokyo, Japan). For tissue samples, glass beads of 35 μm radius were attached to pyramidal cantilevers (MLCT; Bruker, Camarillo, CA, USA) with a spring constant of 0.07 N/m. For polyacrylamide gel verification, pyramidal cantilevers with spring constant 0.05 N/m and a half-angle to face of 17.5° were used. Sensitivity of the cantilevers was calculated by analysing the gradient of the force–distance curve in the AFM software (JPK Instruments) on the base of the petri dish. Tissue samples thawed from frozen or polyacrylamide gels stored in phosphate-buffered saline (PBS) were attached to the base of a petri dish with a cyanoacrylate adhesive. For tissue samples, indentation tests for tissue samples were carried out to generate 30 force curves across 6 regions of 100 × 100 μm^2^ size, with an approach speed of 5 μm/s and a maximum set force of 1 nN. Polyacrylamide gel verification was performed across multiple gels prepared independently, with 9 measurements within a 100 × 100 μm^2^ area, which was averaged to give each independent value to be used in data representation and statistical analysis. The Young’s modulus was calculated using the AFM software by fitting the Hertz contact model^60^ to the acquired force curves.

### Polyacrylamide gels

Fresh 13 mm glass coverslips were activated by dipping in 0.1 M NaOH and left to dry. (3-Aminopropyl) triethoxysilane 4.0% (Sigma-Aldrich, St Louis, MO, USA) was used to coat the coverslips with an amine-reactive film, before washing in distilled water for 10 min. After drying, coverslips were coated with 2.5% glutaraldehyde (Sigma-Aldrich) for 30 min, and then washed twice in distilled water for 10 min, to produce a coating of aldehyde functional groups. Polyacrylamide gels (500 μl) were made by mixing PBS, acrylamide/bisacrylamide (29:1) 40% vol (Sigma-Aldrich) 1 μl TEMED (Sigma-Aldrich) and 2.5 μl 10% ammonium persulphate. The acrylamide/bisacrylamide volume for each gel was 34.9, 59.4 and 125.3 μl, known to produce gels of 1, 4 and 25 kPa stiffness, respectively. In all, 10 μl of gel was applied to dichlorodimethylsilane (Sigma-Aldrich) coated glass microscope slides and activated coverslips were placed on top for 45–60 min. Coverslips were washed in PBS on the rocker for 20 min and then sterilised in fresh PBS for 30 min with ultraviolet light. A total of 50 μl of the heterobifunctional sulphosuccinimidyl 6-(4′-azido-2′-nitrophenylamino) hexanoate (sulpho-SANPAH) was added and photoactivated for 5 min with ultraviolet light. After washing with PBS, coverslips were coated with 10 μl/ml fibronectin solution for 1.5 h and washed before cell seeding. If adrenaline or blebbistatin was to be used, cells were seeded in normal media and left for 1–2 h to spread and attach, before changing the media to drug-containing media.

### Cell culture

BxPC-3 and Suit2-007 cells were obtained from Dr Silvia Ottaviani at Hammersmith Hospital, London, UK. Both BxPC-3 and AsPC-1 cells were grown in RPMI 1640 with 10% foetal bovine serum heat inactivated (Gibco, Loughborough, UK), 2 mM L-glutamine, 1% penicillin/streptomycin (Sigma-Aldrich) and 1 % fungizone Anphotocerin B (Gibco, UK). Suit2-007 cells were grown in Dulbecco’s modified Eagle’s medium with the same supplements added. All cells were tested for mycoplasma contamination.

### Chemoresistance

To assay chemoresistance, cells were seeded on polyacrylamide gels and left to grow for 72 h. After this, media was changed for media with the relevant concentration of gemcitabine hydrochloride (Sigma-Aldrich) or paclitaxel (Sigma-Aldrich) and left for 72 h. Gels were washed with PBS and incubated with 70 μl MTS reagent (Promega, Madison, WI, USA) and 350 μl of clear RPMI media (Gibco, UK) for 1 h. The solution in each well was split into three wells of a 96-well plate and absorbance was read at 490 nm with a Tecan plate reader (Tecan, Grödig, Austria).

### Cell fixation and staining

Cells on coverslips were fixed 72 h after seeding unless otherwise specified, using 4% paraformaldehyde in PBS for 30 min at 37 °C. For staining, cells were permeabilised and blocked with 2% bovine serum albumin/0.1% Triton-X in PBS for 10 min and then stained with primary antibody diluted in 2% bovine serum albumin in PBS for 1 h. After a PBS wash, cells were stained with the relevant secondary antibody (1/500) and phalloidin (1/500) in PBS. Following a further PBS wash, coverslips were mounted in ProLong Gold Antifade with DAPI (4,6-diamidino-2-phenylindole; ThermoFisher Scientific, Eugene, OR, USA). Images were taken with a Nikon Eclipse Ti-E microscope (Nikon, Kingston-upon-Thames, UK) at × 20/ × 40 magnification.

Antibodies used were the following: vimentin (Dako M075, Carpinteria, CA, USA), E-cadherin (Santa Cruz sc-21791), β-catenin (Santa Cruz sc-7199, Dallas, TX, USA), YAP (Santa Cruz sc-101199), TAZ (Abcam ab84927, Cambridge, UK), Phalloidin (Life Technologies A22283, Eugene, OR, USA), AlexaFluor 488 goat anti-rabbit IgG (Life Technologies A11034), AlexaFluor goat anti-mouse IgG (Life Technologies A11029), Goat antibody to rabbit IgG (horseradish peroxidase (HRP)) (Abcam ab6721), and HRP Goat Anti-Mouse IgG (Life Technologies 626520).

### Image analysis

Alignment analysis on collagen images was conducted as described previously for 75 × 75 μm^2^ regions.^33^ Nuclear localisation of β-catenin, YAP and TAZ was calculated as how many cells at × 20 magnification showed staining for the relevant antibody within the nuclear region, as delineated by staining with DAPI. Vimentin and F-actin intensity were determined by corrected total cell fluorescence in ImageJ (NIH, MD, USA), as calculated by 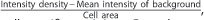
to assess only the fluorescence due to cell-specific staining. Roundness was calculated as a product of the variance of the radius of a given cell, for 360 radii taken at 1° angles, using a custom-built code. Clustering scores were determined by inspection of images with phalloidin staining, with the non-clustering percentage calculated as the percentage of cells that did not form any cell–cell junctions.

### Western blottings

Cell lysates from cells grown on polyacrylamide gels of different rigidities were prepared with radio immunoprecipitation assay buffer (Sigma, R0728, Berlin, Germany) with protease and phosphatase inhibitors (Sigma P4340). Protein quantification was determined with the DC Protein Assay (Bio-Rad, 500-0113, Hercules, CA, USA) according to the instructions provided. Sodium dodecyl sulphate–polyacrylamide gel electrophoresis was used to separate samples under reducing conditions and were transferred to a polyvinylidene difluoride membrane (Protran BA85, Berlin, Germany) and blocked with 5% bovine serum albumin (Sigma, A8022) in TBST (TBS with 0.1% Tween-20 (Sigma, P1379)). Primary antibodies were prepared in this blocking solution and membranes were incubated overnight at 4 °C. The membrane was washed in TBST and incubated with HRP-conjugated secondary antibodies in TBST for 1 h at room temperature. The membrane was washed and developed with the HRP substrate (Millipore, WBLUR0100, Billerica, MA, USA).

### Reverse transcriptase–PCR

Total RNA was extracted using the RNeasy Mini Kit (Qiagen, 74104, Hilden, Germany) and 1 mg of total RNA was reverse-transcribed using the High-Capacity RNA-to-cDNA Kit (Applied Biosystems, 4387406, Loughborough, UK) according to the manufacturer’s instructions. Quantitative PCR was performed using the SYBR Green PCR Master Mix (Applied Biosystems, 4309155) with 100 ng cDNA input in 20 ml reaction volume. GAPDH (glyceraldehyde 3-phosphate dehydrogenase) expression level was used for normalisation as a housekeeping gene. The sequences were as following: vimentin: forward-5′-GGAAACTAATCTGGATTCA-3′, reverse-5′-CATCTCTAGTTTCAACCGTC-3′ E-cadherin: forward-5′-CCGAGAGCTACACGTTC-3′, reverse-5′-TCTTCAAAATTCACTCTGCC-3′ CTGF: forward-5′-TTAAGAAGGGCAAAAAGTGC-3′, reverse-5′-CATACTCCACAGAATTTAGCTC-3′ ANKDR1: forward, 5′-TGAGTATAAACGGACAGCTC-3′ and reverse, 5′-TATCACGGAATTCGATCTGG-3′ and GAPDH: forward-5′-ACAGTTGCCATGTAGACC-3′, reverse-5′-TTTTTGGTTGAGCACAGG-3′. All primers were used at 300 nM final concentration. The relative gene expression was analysed by comparative 2^−ΔΔct^ method.

### Statistics

Unless otherwise specified, statistical analysis was conducted as an unpaired Mann–Whitney test (two-tailed). All statistical tests where a normal distribution was assumed had no significant differences in variance.
