# Supplementary material for: Matrix stiffness induces epithelial–mesenchymal transition and promotes chemoresistance in pancreatic cancer cells
Source: Oncogenesis. 2017 Jul 3;6(7):e352–. doi: 10.1038/oncsis.2017.54 (PMC5541706; doi:10.1038/oncsis.2017.54)
Supplement: Supplementary Figures [file oncsis201754x1.pdf]

Supplementary Figure 1

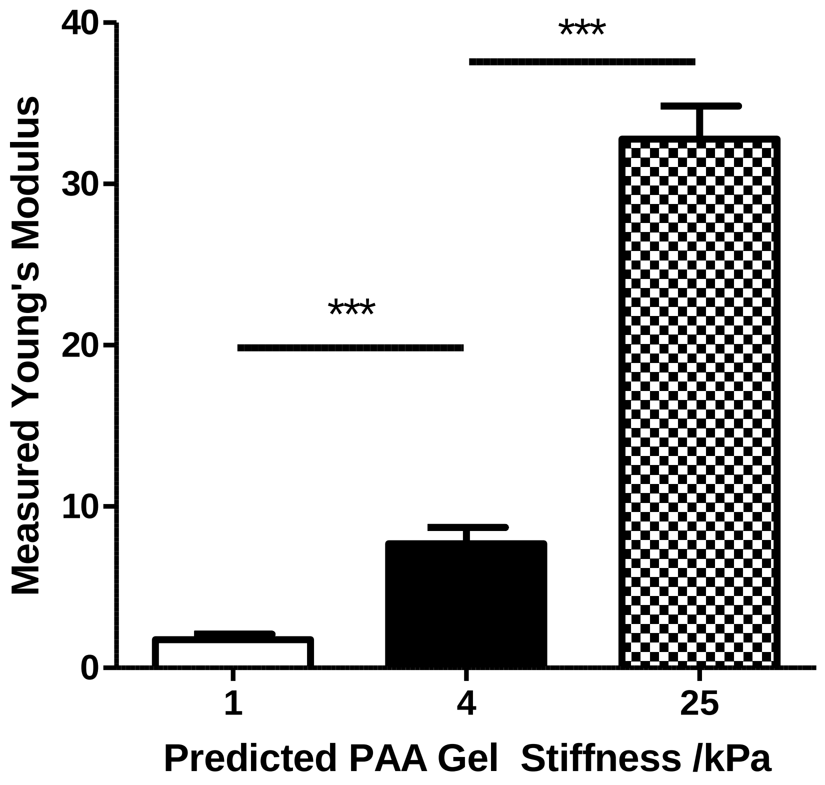

Supplementary Figure 1: Stiffnesses of polyacrylamide gels measured by AFM. Atomic force microscopy analysis of fabricated polyacrylamide gels to verify changes in stiffness. For predicted polyacrylamide stiffnesses of 1, 4 and 25 kPa, n = 8, 14, 10 gels respectively. Values represent mean ± s.e.m. ***$P < 0.001$ for unpaired Mann-Whitney test (two-tailed)

Supplementary Figure 2

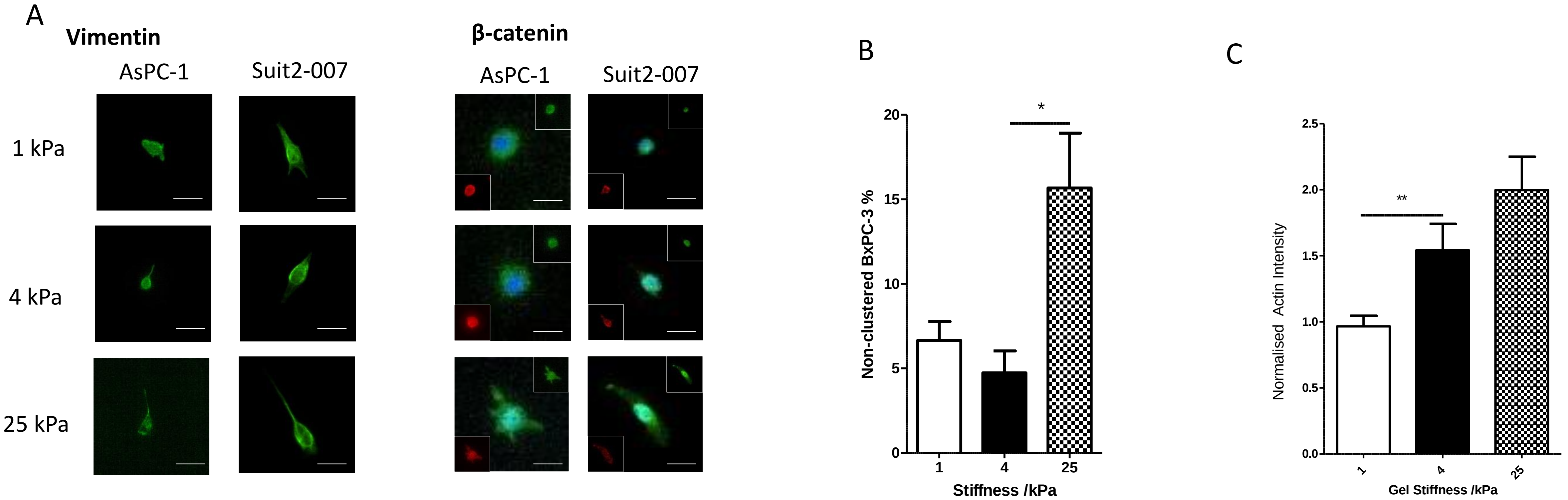

Supplementary Figure 2. Role of matrix stiffness in EMT induction in AsPC-1 and Suit2-007 cells. A) Vimentin, E-cadherin, and β-catenin immunofluorescence images of AsPC-1 and Suit2-007 cells on matrices of varying stiffness. For β-catenin, main image shows merge of marker staining (green) and DAPI (blue), top right inset is marker staining, and bottom left inset is phalloidin staining (red) of actin cytoskeleton to show cell shape. Scale bar = 50 μm. **B)** Percentage of non-clustered BxPC-3 cells per region grown on matrices of varying stiffnesses. 1 kPa, n = 25; 4 kPa, n = 24; 25 kPa, n = 23 regions. Values represent mean ± s.e.m. *P < 0.05 for unpaired Mann-Whitney test (two-tailed) **C)** F-actin intensity (Corrected Cell Total Fluorescence) with phalloidin staining. For 1,4, and 25 kPa respectively, n = 52,26,33 cells. Values represent mean ± s.e.m. **P < 0.01 for unpaired Mann-Whitney test (two-tailed)

Supplementary Figure 3

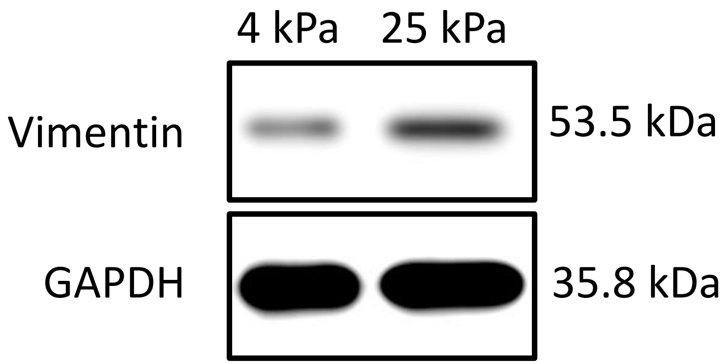

Supplementary Figure 3. Vimentin expression increases with stiffness. Western Blot for Vimentin expression, indicating an increase in expression with stiffness.

Supplementary Figure 4

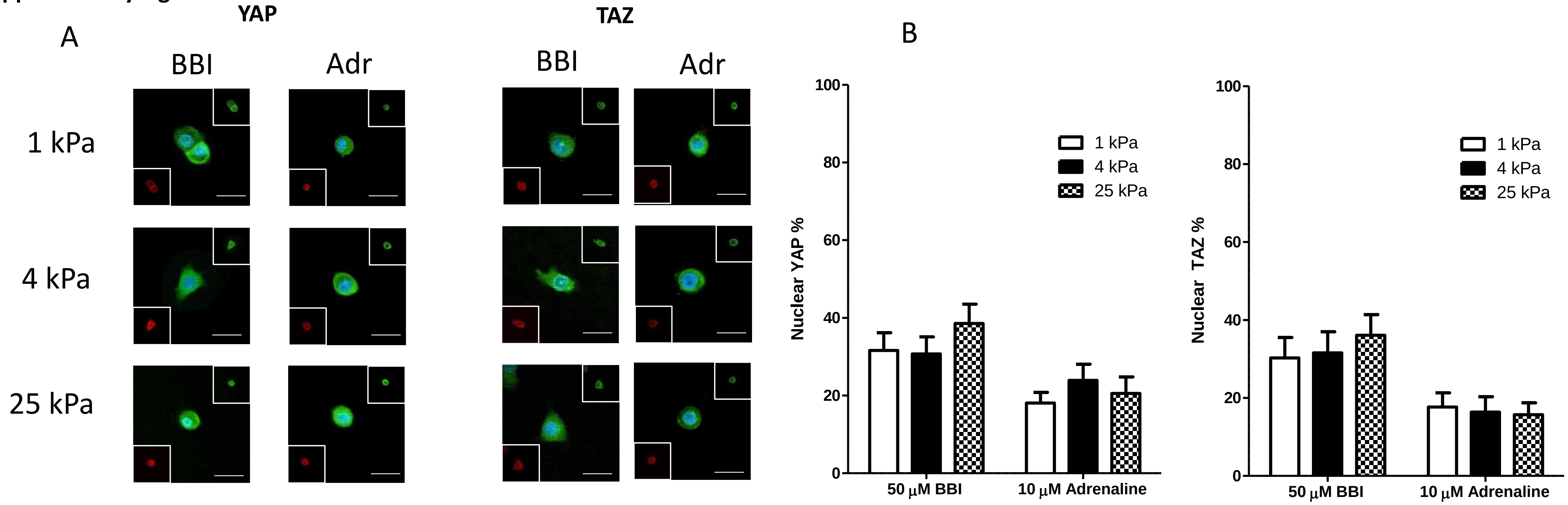

Supplementary Figure 4. Role of matrix stiffness on YAP/TAZ nuclear localisation with disrupted signalling. A) Changes in YAP and TAZ localisation to the nucleus in BxPC-3 cells in the presence of 50 µM blebbistatin (BBI) or 10 µm Adrenaline (Adr). Scale bar = 25 µm. **B)** Nuclear Localisation Percentage of YAP in BxPC-3 cells. For 1,4, and 25 kPa respectively, 50 µM BBI, n = 17, 18, 14 regions; 10 µM Adrenaline, n = 11, 9, 12 regions. Nuclear Localisation Percentage of TAZ in BxPC-3 cells. For 1,4, and 25 kPa respectively, 50 µM BBI, n =16, 19, 16 regions; 10 µM Adrenaline, n = 11,11,9 regions. Values represent mean ± s.e.m for unpaired Mann-Whitney test (two-tailed)
